# Rice Starch Chemistry, Functional Properties, and Industrial Applications: A Review

**DOI:** 10.3390/polym17010110

**Published:** 2025-01-03

**Authors:** Rizwan Shoukat, Marta Cappai, Luca Pilia, Giorgio Pia

**Affiliations:** Department of Mechanical, Chemical, and Materials Engineering, University of Cagliari, Via Marengo 2, 09123 Cagliari, CA, Italy

**Keywords:** starch chemistry, tailoring and isolation, green polymers, functional features, industrial applications

## Abstract

Starch is among the most abundant natural compounds in nature after cellulose. Studies have shown that the structure and functions of starch differ extensively across and among botanical types, isolation procedures, and climate factors, resulting in starch with significant variations in its chemical, physical, morphological, thermal, and functional characteristics. To enhance its beneficial properties and address inherent limitations, starch is modified through various techniques, resulting in significant alterations to its chemical and physical characteristics. These structural modifications impart considerable technological and industrial versatility. In the food sector, modified starch serves as a thickener, shelf-life extender, fat replacer, texture modifier, gelling agent, and stabilizer. In non-food applications, it functions as a sizing agent, binder, disintegrant, absorbent, and adhesive and is employed in construction as a sealant and to improve material bonding strength. The demand for modified starch has surpassed that of its native counterpart, reflecting its growing market value and the industry’s interest in products with novel functional attributes and enhanced value. This study focuses on rice starch, highlighting its structure and composition and their impact on physicochemical properties and functionality. Additionally, it examines the enhancement of its techno-functional characteristics, achieved through various modification processes.

## 1. Introduction

Starch is a biodegradable and cost effective polymer widely found in cereal grains, roots, tubers, fruits, and leaves and serves as the primary source of nutrition, comprising 60–70% of the diet for over 60% of the people worldwide [1,2,3]. The primary industrial sources of starch are rice, cassava, wheat, potatoes, and maize. Starch’s nutritional composition emphasizes its importance due to it being an essential mineral and carbohydrate source; starch contains vital micronutrients, including copper, iron, and magnesium, alongside phosphorus. Starch, serving as the main storage carbohydrate in plants also holds significant importance as a fundamental agricultural resource for humanity [4,5]. Each year, rice and cereals, acting as primary starch roots for human consumption, collectively yield two gigatons thereabouts. In established and industrialized territories, starch typically comprises, at the smallest amount, 35% of everyday caloric consumption. In numerous regions, particularly the Eastern world (China, Japan, Korea, and Australia) and African states (Nigeria, Guinea, and Mali), starch can contribute up to 80% of daily caloric intake, often deriving from a singular staple crop such as rice [6,7]. Rice starch exhibits greater diversity compared to other cereal grains, which is significant as it enables the isolation of starch with a wide range of functionalities. New rice cultivars are continually being developed, with the total number now exceeding 2000 worldwide [8,9]. The optimized composition of rice starch is responsible for its smooth texture, favorable amylose-to-amylopectin ratio, mild flavor, white color, hypoallergenic properties, high digestibility, strong consumer acceptance, small granules, increased paste freeze–thaw stability, and high acid resistance. Starch is widely utilized due to its abundant availability, versatility, and adaptability to various processes [10]. These distinctive properties have driven the increased demand for rice starch across various industries.

**Figure 1:**
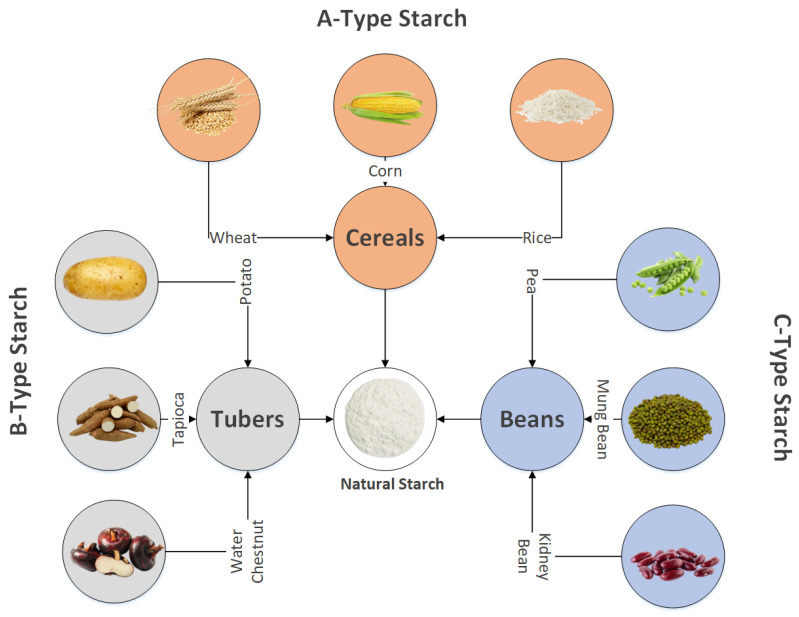
Starch source diversity in plant species.

However, natural plant-derived starch often fails to withstand extreme processing conditions, such as high shear rates, prolonged exposure to strong acids and alkalis, or repeated freezing and thawing cycles, making it unsuitable for many industrial applications. To resolve these challenges, various modification techniques are employed to enhance or alter its inherent properties and introduce specialized characteristics that meet industrial requirements [11,12]. Key modification methods include chemical processes like oxidation, esterification, etherification, and hydroxypropylation; enzymatic processes like dextrinization; and physical methods such as high-pressure treatment and extrusion. These modifications enable the production of starch with tailored properties for specific applications [13,14]. Despite being widely consumed in unprocessed forms, like cereal grains (rice) and potatoes, starch is becoming more and more prevalent in processed foods. Starch is used in various food products as a thickening, binding, encapsulating, stabilizing, and gelling agent in a vast range of diets, such as soups, baby foods, cooked items, puddings, candies, ice cream, meat products, sauces, snack foods, and soft drinks [15,16,17]. Starch is also an essential substrate for the synthesis of many products, such as glucose syrups and maltodextrins [18]. Besides its importance in food products, starch is widely utilized across a range of non-food sectors due to its functionalities, non-toxic characteristics, and biodegradability. These include agricultural industrial chemicals, sealants, beauty aids, cleansing agents, medicine, oil earth boring, thin paper making, plastics, and fabrics. Within these industries, the distinctive ability of starch is greatly valued and actively utilized [19]. The different sources of starch are shown in Figure 1.

The aim of this study is to provide an in-depth analysis of the various components and chemistry of rice starch, focusing on their influence on physicochemical properties and functional characteristics, which are comprehensively examined. It also examines various starch tailoring and isolation techniques. The impact of modifications in the molecular structure and amylose–amylopectin ratios influence critical properties, such as gelatinization, retrogradation, and pasting properties, are explored. Furthermore, the review highlights emerging innovation in chemical treatments for starch modification, emphasizing how these techniques fine-tune operational aspects of starch to enhance its performance and utility in diverse industrial applications in food and non-food sectors.

## 2. Starch Chemistry

Starch, referred to as amylum, has multiple applications in food, clothing, refined chemicals, paper production, farming, petroleum, and building engineering. In 2019, the industrial demand for starch was estimated to have reached USD 87.93 billion [20]. The worldwide starch industry is predicted to produce revenues of USD 112 billion in 2024, with a CAGR (compound annual growth rate) of about 5.9% for the projected time frame from 2019 to 2024 [21].

**Figure 2:**
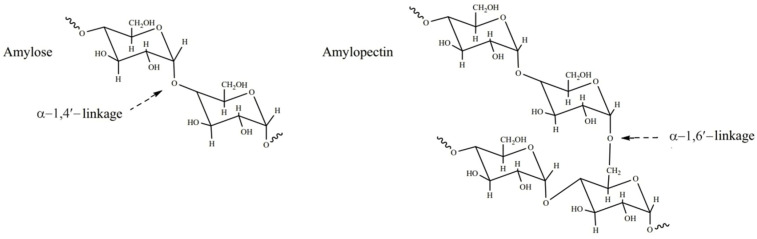
Molecular form of starch (amylose together with amylopectin).

Rice starch comprises two forms of α-glucans, namely amylose and amylopectin (refer to Figure 2). These polymeric compounds are stored in plant structures as starch granules (SGs), exhibiting variations in size and shape across different species. Granules integral to starch’s structure are specialized and unique particles. Rice starch granules stand out among cereal grains as the smallest, displaying angular and polygonal shapes typically within the size range of 2 to 7 μm. Essential chemical and physical attributes, such as the mineral content, ratio of amylose/amylopectin, granule mean size, and distribution, significantly influence the properties of starch [22]. The complex processing of starch leads to inherent variability in structures of amylose and amylopectin molecules, resulting in diverse granule morphologies. Interestingly, significant differences in granule form and size are connected to a variety of functional features in various nutritional systems or depending on the botanical origin. There can be additional connections between granule form, nutritional properties, and manufacturing procedures. The resistant starch (a form of starch that resists enzymatic digestion in the small intestine and is subsequently fermented in the colon), characterized by its granule structure that enhances resistance to digestion, is beneficial for gut health, being resistant in food processing due to lower swelling and solubility with an elevated gelatinization temperature [23]. The starch constitutes between 98 and 99% of the dry weight of the SG. Many authors have provided in-depth descriptions of the composition and characteristics of amylose and amylopectin. Despite experimental evidence supporting the existence of some amylose sequences branching, amylose is primarily considered to be a polymer (straight chain) of glucose α-1,4-linked molecules [24,25,26].

**Figure 3:**
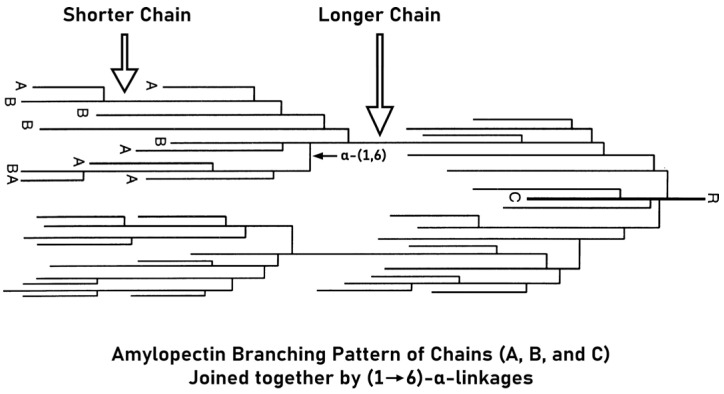
A cluster framework for structuring the amylopectin sequence: (A–B) describes the exact positions of A–B series in a cluster, whereas (C) represents the C-series position in the molecular structure (reprinted with permission from Oxford university Press Wang and Bogracheva [40]).

The exact positioning of amylose within a SG continues to be a topic of discussion. Multiple potential locations have been suggested, such as (i) within the unformed lamellae, (ii) existing in shapeless growing bands, or (iii) intermixed or amylopectin co-crystallization [27]. Amylose is hydrophilic because it takes on a helical structure and contains hydrogen atoms within. It is because of this property that it can form clathrate complexes, in which a host molecule encases a guest molecule. Untied fats and glyceride units, particularly liquor with iodine, can all be involved in these mixtures [28,29]. According to Takeda and coworkers [30], amylose extracted from starchy rice exhibits average polymerization degree of weight (DPw) and average polymerization degree of number (DPn) values ranging from 2750 to 3320 and 980 to 1110, respectively, along with an average chain length (CL) of 250 to 370. An analysis of iso-amylase (an enzyme that converts branched amylopectin and glycogen into linear molecules like amylose) from six white rice varieties was conducted using high-pressure size-exclusion chromatography coupled with a refractive index detector and multi-angle laser light-scattering detector (HPSEC-MALLS-RI). For the analytical procedure, a guard column in conjunction with two serially connected size-exclusion chromatography (SEC) columns was employed, operating under controlled conditions at 70 °C. Sample separation was conducted with detection facilitated by multi-angle laser light scattering (MALLS) and differential refractive index (RI) detectors. The efficacy of the SEC columns was assessed by measuring the theoretical plate count, utilizing fructose as the standard analyte. Calibration of the RI detector was performed using a series of sodium chloride (NaCl) standards. The 90° photodiode of the MALLS detector was calibrated with high-performance liquid chromatography (HPLC)-grade toluene, while the other 17 photodiodes at multiple scattering angles were standardized relative to the 90° photodiode using a dextran reference (Dextran 25,000). The volumetric delay between the MALLS and RI detectors was accurately determined using bovine serum albumin as the calibrant. The findings revealed molecular mass (weight mean) (M_w_) and molecular mass (number mean) (M_n_) values varying between 5.1 to 6.9 × 10^5^ g/mol and 1.4 to 1.8 × 10^5^ g/mol [31]. The amylose content differs altogether across rice cultivars. For example, glutinous (waxy) rice starch usually contains no more than 1.3% amylose, while non-glutinous rice starch can contain up to 37% amylose. The alpha-amylose centralization of processed grain is generally described as flex (waxy) within 1–2%, low at 7–20%, average up to 25%, or above average (>25%) [32]. The combination of amylose and iodine results in a blue-black coloration of amylose. This color change forms the footing of frequently employed colorimetric techniques for working out the amylose portion of a test [33]. Amylopectin, the major constituent of common starches, comprises approximately 70–85% of their composition. This highly branched polysaccharide is characterized by its heterogeneous structure, consisting of three distinct chain types: A, B, and C. A-chains, linked to either B- or C-chains through α-(1→6) linkage glycosidic bonds at their reducing terminal, are distinguished by their lack of branching. In contrast, B-chains exhibit a branched structure, forming linkages with other B- or C-chains and possessing branch points where A-chains or other B-chains are attached via α-(1→6) linkages to the O-6 position of glucosyl residues. Importantly, each amylopectin molecule contains a single C-chain, which carries the sole reducing end of the molecule (see Figure 3) [34,35,36]. A-chains, typically composed of 6 to 15 glucose units, establishing the backbone of the molecule. Based on their length and the number of clusters they span within the amylopectin molecule, B-chains are broadly classified into four groups: B1, B2, B3, and B4. B1 and B2 chains, generally spanning one to two clusters, exhibit shorter chain lengths. Conversely, B3 and B4 chains, capable of traversing up to four clusters, possess longer chain lengths, amplifying the molecule’s overall structural complexity [37].

Amylopectin is listed as a α-1,4-linked glucose molecule, with approximately up to 6% being an α-1,6-assembly at the branch positions, resulting in a high degree of branching [38]. HPSEC-MALLS-RI studies reveal that the M_w_ obtained from numerous plants for amylopectin has been testified to be between 0.7 × 10^8^ and 57 × 10^8^ g/mol. Amylopectins from waxy starches have higher molecular weights than those from normal starches, reflecting a more complex structure with more branched chains. Waxy amylopectins also exhibit greater molecular densities due to the absence of long chains found in normal amylopectins, leading to a more compact molecular arrangement [39]. Starch particles have a semicrystalline nature because they contain both crystalline and non-crystalline parts. This semicrystalline structure results from the organized arrangement of starch molecules in a radial pattern. SGs are labeled as semicrystalline due to their combination of crystalline and amorphous regions [11].

Starch contains amylopectin, which uniquely allows the outer sequences of amylopectin and amylose to create helices, which merge to create crystalline regions. The crystallinity seen in amylopectin-rich starches comes from these helices within the outer sequences of amylopectin. In waxy and regular starches, amylose does not have a significant effect on crystallinity. However, in high-amylose starches, its influence can be notable [41,42]. Starches from corn, potatoes, tapioca, and wheat have been found to contain between 32 and 64% double-helical material [43,44,45]. In contrast, it was experimentally investigated that 63% of double-helical amylopectin was present in waxy rice starches [46]. Amylopectin structures of Indica and Japonica waxy varieties reveal distinct variations in their chain length distributions, degree of polymerization, and branching patterns, as indicated by the ratios of different chain types (F1, F2, and F3). Indica amylopectin exhibits an average CL of 21–22 glucose units, while Japonica waxy amylopectin displays a slightly shorter average CL of 19–20 glucose units. The DP, representing the average number of glucose units per amylopectin molecule, is notably higher in Japonica waxy varieties (8.2–12.8 × 10^3^) compared to Indica (4.7–5.8 × 10^3^), indicating a larger molecular size. Branching patterns, represented by the ratios of F1, F2, and F3 chains, also differ between the two varieties. Indica amylopectin shows a higher proportion of shorter chains (F2 and F3) relative to longer chains (F1), while Japonica waxy amylopectin exhibits a higher proportion of longer chains (F1). These structural differences are further corroborated by the iodine-binding capacity, which is lower in Japonica waxy varieties, reflecting its reduced amylose content and higher amylopectin content with longer chain lengths. The phosphorus content, primarily in the form of glucose-6-phosphate, is relatively similar between the two varieties. Beta-amylolysis, an indicator of amylopectin’s susceptibility to enzymatic degradation, is comparable between Indica and Japonica waxy varieties. Table 1 illustrates how the characteristics of amylopectin are influenced by the grain cultivar for various types of rice. To increase the number of applications for starch in food, a number of isolation techniques have been used. Nevertheless, despite their great versatility, native starches are less often used in the food processing industry because of a few physical and chemical flaws, including a high tendency toward retrogradation, low thermal stability, low shear resistance, and degradation [47,48]. To attain the desired techno-functional properties, rice starch can undergo modification using various chemical, physical, and genetic techniques. Chemical modifications are commonly utilized to address the inherent variability and limited adaptability of starch to diverse processing conditions [49]. When rice starch is chemically modified, its freeze–thaw steadiness, emulsifying qualities, gel syneresis, and gelling competence are typically reduced [31].

### 2.1. Rice Starch Tailoring

The primary carbohydrate found in human diets and considered the fundamental energy source for physical development and growth is starch. Amylose, along with amylopectin, other trace molecules, and ash are the main constituents of starch. The starch chemical composition was found to vary significantly (see Table 2). The insignificant elements determine the starch purity; lower values indicate higher starch purity. These elements are different based on the quantification techniques and starch isolation methods. The typical moistness and protein and fat contents of the starch fractions are up to 10.62%, 0.52%, and 0.67% respectively, as stated by [50], and according to Ashogbon’s [51] findings, are from 10 to 12%, from 0.1 to 0.70%, and from 0.4 to 0.43%, respectively. However, the starches that were isolated from four distinct rice cultivars had fat and protein contents that varied from 0.1 to 0.7% and 0.1 to 0.36%, respectively [52]. The ability of lipids to form complexes with amylose allows for the modification of certain efficient features of starch. For instance, the formation of lipid–amylose complexes reduces the rate of rice starch hydrolysis, its solubility in water, its ability to swell, and the solubility and mobility of amylose to form crystals and double helices [53,54]. The starch granule-associated proteins (SGAPs) are located both internally and on the surface of starch granules [55]. The extraction of the former proteins requires granule swelling and employs powerful detergents, while the proteins on the surface can efficiently be extracted using a buffer solution. Through a decrease in the viscosity of the developing pastes, the SGAPs can affect the starch pasting characteristics. Nevertheless, some SGAPs, such as starch rice granule-bound synthases, increase the stiffness in SGs, which lessens shear-triggered starch disintegration [56]. Significant differences in protein expression, such as ribosomal protein and 14-3-3-like protein, were found in ten kinds of extracted SGAPs. However, their absence may have a substantial impact on the levels of resistant, slowly digestible, and quickly digestible rice starch [57].

### 2.2. Rice Starch Isolation

In terms of grains’ overall composition, starch is the most abundant component and constitutes the highest proportion. The isolation process for rice grain starch differs significantly from that of other components. Because rice starch has a distinct and unique protein composition, different methods are used for its isolation.

**Figure 4:**
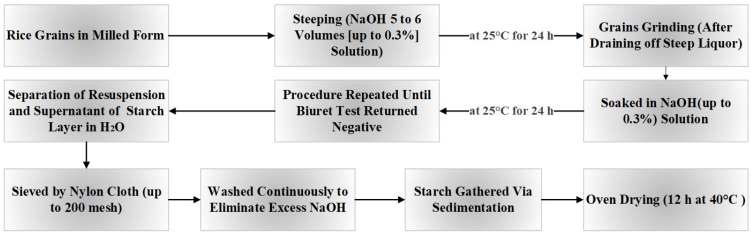
Flowchart: extraction approach for starch (adopted and modified from Verma D.K. and Srivastav P.P. [1]).

In the rice case, separating starch from other components, such as fat, fiber, and protein, has led to the evolution of the method for isolating rice starch. The avoidance of amylolytic damage in grains, their gelatinization, efficient deproteinization, and decreasing the loss of small SGs should, therefore, be the main areas of attention [64]. The protein composition of rice includes the following components in the following proportions: 5%, 3%, 80%, and 12%, respectively, of albumin, prolamin, glutelin, and globulin. These protein components dissolve easily in different solvents/solutions. Examples include albumin in water, prolamin in ethanol, globulin in salt solution, and glutelin in alkaline solution [30]. Henceforth, the primary technique utilized in starch isolation involves alkali extraction [56,57]. The protein composition in rice starch varies significantly across different rice varieties and is also influenced by the purification process and the overall quality of the starch. In Figure 4, a precise presentation of the flow diagram illustrating the alkaline-based method of separation is presented [1].

Grains are steeped in a 0.3% sodium hydroxide solution at 25 °C for 24 h. Following steeping, the softened endosperms are gently ground, and the resulting slurry is diluted, stirred, and left to settle overnight to facilitate starch separation. The washing process is repeated until the supernatant is clear and free of protein, which is confirmed using the biuret test. The starch is then filtered through a fine nylon cloth to remove impurities, washed to eliminate residual alkali, and collected via sedimentation and dried at 40 °C [65]. The segregation of starch from rice also involved the use of a 0.35% NaOH solution via the alkali extraction method [66]. The starch layer was re-slurried with H_2_O and filtered after the rice powder (flour) was immersed in NaOH solution and stored at 4 °C for two days. The remaining fluid was appropriately disposed of. After filtering, the slurry was allowed to settle at 4 °C for 24 h to remove the residue that remained. The starch was then dried in a hot microwave at 40 °C for 24 h. Rice starch isolation has traditionally been accomplished through the alkali isolation method; however, this approach generates large amounts of alkaline waste and is, therefore, inappropriate [67]. Moreover, a comparison between the alkali and other isolation techniques has been studied. The four isolation techniques employed by Zhong et al. were protease, NaOH 0.1%, NaOH 0.4%, and 1% sodium dodecyl sulfate (SDS) [68]. Based on the isolation techniques used, rice starch was isolated for the study and its pasting and rheological properties evaluated. Comparing protease and NaOH (0.1%) techniques to the treatment with NaOH (0.4%) and SDS, reduced elastic moduli, peak pasting temperatures, and stresses were studied in the non-sticky starch paste batches. But the elastic moduli of sticky starch paste were unaffected by the isolation techniques in any appreciable way. However, the protease–starch paste did have a greater yield stress and lower shear viscosity than pastes made using alternative techniques. Similar outcomes were noted in the evaluation of functional characteristics of starch extracted from long-grain rice using alkali and neutral protease treatments. Specifically, alkali-treated starch exhibited a higher swelling rate compared to enzymatically treated starch, although the maximal viscosity pasting decreased [69]. Wang identified an alternative way (high-intensity ultrasound coupled with SDS or alone) for rapid rice starch separation that eliminates the need for any chemical agents, unlike the conventional alkaline-based route. The sonication system does not affect the amylose content or thermal aspects. Additionally, scanning electron microscopy (SEM) revealed that sonication had not caused any alterations to the starch surfaces [70]. The freeze–thaw infusion method has been applied recently in intriguing work to improve the extraction efficacy of starch from breached rice. This process makes it easier for enzymes to enter substrates quickly, which increases the efficiency of enzymatic reactions both favoring and independent of the substrate surface. The starch extraction yield of the food-grade protease-related freeze–thaw infusion method was higher at 69.31% compared to the alkali treatment and protease treatment alone, which achieved extraction efficiencies of 66.81% and 61.74%, respectively. Moreover, the pasting, thermal features, and shape of the isolated rice starches were not affected by the extraction procedure [71]. Thus, the freeze–thaw infusion process presents itself as a viable and effective substitute for the conventional alkali approach in extraction.

### 2.3. Rice Starch Variation and Modification

To optimize its working features for use in food, starch can undergo modifications via physical, chemical, or enzymatic approaches. These processes modify the physicochemical properties of natural rice starch [6]. Many modern approaches, including dual modification, oxidation, etherification, cross-linking, and esterification, are employed to amend the physical characteristics and starch thermal transition behavior, with cross-linking agents being the most significant technique. However, the plant origin of the starch and the cross-linking agent used determine the cross-linking and its effects. The cross-linked starch exhibited a reduced retrogradation rate and elevated gelatinization temperatures, attributed to decreased amorphous chain mobility within the starch granule due to the formation of intermolecular bridges [72]. Acetic anhydride, anhydrides of adipic and ethylic acids, oxyhalide of phosphorus (POX_3_), STMP (Na_3_P_3_O_9_), STPP (Na_5_P_3_O_10_), ethylene oxide, and oxolane-2,5-dione are, in general, utilized foremost in chemical amendment procedures. Other treatments include esterification and chemical reactions with H_2_SO_4_ and HCl; a H_2_O_2_ bleaching process, KMnO_4_, and hypochlorite (NaOCl) oxidation; and other treatments employing various combinations of these chemical processes [73].

#### 2.3.1. Rice Starch Cross-Linking: Exploring Bond Formation

The SG resists heat, shearing, and acids more effectively as a result of the cross-linking process [74]. Cross-linking refers to the formation of covalent bonds between starch molecules by targeting the hydroxyl groups present on amylose and amylopectin chains, facilitated by specific cross-linking agents. Quite a few agents (chemicals), including acetic–adipic combined anhydrides, epichlorohydrin (ECH), POCl_3_, STPP, STMP, and a mixture of STMP and STP, are used in the cross-linking process to modify the native rice starch. Nevertheless, the U.S. no longer uses ECH for food-grade purposes because chlorohydrins are known to be carcinogens. It is worth noting that because of the molecular assemblies of cross-linked coordination, the nature of the cross-linking driving force primarily influences the functional properties of modified rice starch [34]. Because of intermolecular links, cross-linked starch exhibited a lowered retrogradation speed and increased gelatinization temperature, which are related to the reduced mobility of amorphous sequences in SGs [75]. Moreover, cross-linking resulted in improved pasting clarity and increased starch swelling power, while concurrently reducing the apparent content of amylose [76]. According to [77], POCl_3_-induced cross-linked SGs showed reduced solubility and freeze–thaw stability but an increase in the gelatinization temperature and shear stability. Conversely, [78] observed a decrease in the gelatinization temperature of cross-linked rice starch compared to the native equivalent. This is explained by the fact that there was less gelatinized starch remaining after cross-linking. It is true that cross-linked sticky and non-sticky starches have divergent functions; non-sticky starch showed negative effects, while waxy starch experienced an increase in the pasting temperature and gelatinization via cross-linking treatment. However, the cross-linking process for both waxy and non-waxy rice starches resulted in decreased swelling power and solubility while enhancing shear stability [79]. Cross-linking agents enhance rice starch stability and resistance to heat, acid, and shear by forming covalent bonds between molecules, although they may reduce solubility. In contrast, ethylene oxide introduces hydroxypropyl groups, improving the hydrophilicity, solubility, and freeze–thaw stability while reducing retrogradation. Thus, cross-linking is best for structural integrity, while hydroxypropylation improves solubility and reduces gel hardening.

#### 2.3.2. Rice Starch Oxidation

When a certain amount of an oxidant reacts with starch at a controlled pH and temperature, starch gets oxidized. The industrial production of oxidized starch, which has uses in the culinary field and auxiliary allied fields involving gluing and film formation, is facilitated by the usage of hypochlorite, one of several oxidizing agents. Oxidized starch was prepared by suspending starch (100 g, dry basis) in distilled water (150 mL) at 30 °C, adjusting the pH to 8.5 with NaOH, and adding sodium hypochlorite (2.5% *w*/*w*) while maintaining the pH with H_2_SO_4_. After 30 min, the slurry pH was adjusted to 6.5–7.0 and then centrifuged, washed, and dried at 45 °C for 48 h. Oxidized, cross-linked rice starch was first cross-linked with epichlorohydrin (0.3%, *w*/*w*), followed by NaOCl addition and a pH adjustment to 8.5. Cross-linked, oxidized rice starch was prepared by oxidizing starch with NaOCl, adjusting the pH to 10.0 with NaOH, and then cross-linking with epichlorohydrin. Both products were recovered by centrifugation and drying [80]. Oxidized starch is useful as an outer sizing agent and coating adhesive and can be added to foods with primary flavors like mayonnaise, salad dressing, and lemon curd [16]. An and King [81] discovered that the pasting characteristics of rice starch treated with ozone (O_3_) and starch oxidized with minimal concentrations of chemical oxidizing compounds were similar. Due to hypochlorite generating substantial alkaline wastewater and producing minimal oxidized starch by degrading starch into low-molecular-weight molecules, it is preferable to use environmentally friendly oxidants such as ozone.

### 2.4. Rice Starch Dual Modification

Enhancing the desired properties of starch can be achieved through dual-modification techniques, specifically oxidation and cross-linking. Research has already explored these methods on starches from corn, potatoes, sago, tapioca, and wheat [48]. There have been several studies on the physicochemical properties of controlled modified rice starches reported [74,82]. Undoubtedly, modified starches possess the necessary functional features for food handling; however, they also possess certain unwanted attributes. Additionally, the cross-linking shows a higher tendency toward retrogradation. Both the shear resistance and starch paste viscosity are greatly reduced by oxidation [83]. Hence, chemical and other forms of dual modification play a crucial role in enhancing starch characteristics, thereby improving their functionality for numerous applications.

Ethanoylation/oxidization, cross-linking/basic esterification, or cross-linking/ethanoylation are the primary examples of the dual chemical modifications captured by the systems of dual modifications, which incorporate enzymatic and physicochemical methods [84,85]. Better freeze–thaw stability was achieved by chemically modifying starch through cross-linking and phosphorylation [51,86]. Recently, sodium hypochlorite was used to oxidize rice starch, and epichlorohydrin was used to cross-link it. However, the oxidation and cross-linking processes were unable to significantly alter the shape of the SG. Compared to raw starch, the cross-linked and oxidized starch showed decent paste transparency, lower solubility, and reduced swelling power. Among oxidized cross-linked, native, oxidized, and cross-linked starch, the oxidized, cross-linked starch exhibited the lowest retrogradation tendency and the highest shear resistance. These findings imply that the undesired characteristics of native, oxidized, and cross-linked rice starch might be mitigated by dual modification [80,87].

## 3. Morphological Characteristics

**Figure 5:**
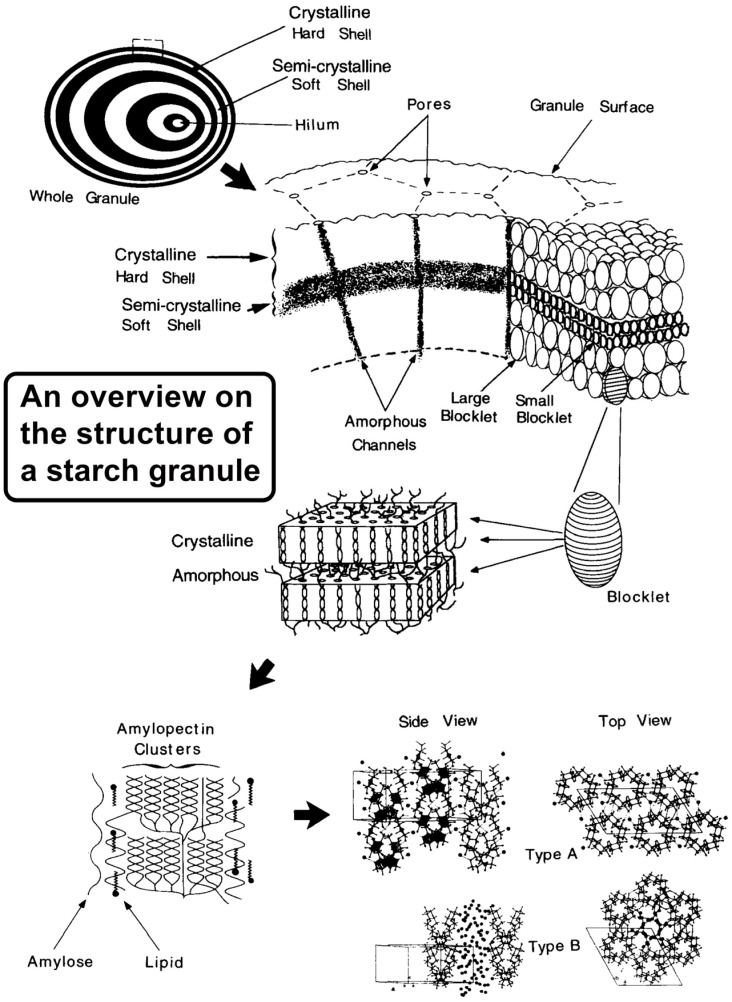
A visual representation showing the arrangement of a SG (reprinted with permission from Elsevier Gallant, Bouchet, and Baldwin [93]).

The SEM-identified morphological development attributes of rice SGs present an understanding of the correlation between rice starch gene variants and fragment shapes [88]. However, another significant technique that has been adopted to investigate the anatomy of starch is laser diffraction. Table 3 presents the geometry and dimensional aspects of rice starches. The polyhedral (PH) form, alongside the precise curves and sides devoid of any openings, were seen in different varieties using light and SEM (such as smooth, uneven, sharp, irregular, and asymmetrical patterns). A single peak mode (unimodal) in the size pattern was observed in all breeds [89]. The overall dimension and form of SGs differ based on the breed and source of each variety [90]. The study by Cai et al. [89] found asymmetrical, sharp curves, having a PH geometry with no surface flaws in grains. Gani et al. [52] found that the SG had an asymmetrical look with a PH geometry. Moreover, a few of the SGs had a spherical pattern. As reported by [91], an SG has an asymmetrical PH geometry. These sphere-structured granules have a polygonal circular form that is bound rigidly and has a relatively clean surface. Multiple types of rice had SG lengths ranging up to 6.7 μm. The environment, farming strategies, breeding variability, and the conversion of starch all contribute to grain particle uniqueness [92]. The maximum dimension size was observed up to 8 μm. A visual representation of the crystal structure of SG is shown in Figure 5 (the scheme is reproduced from [93]). In Figure 5, starch granules display alternating semicrystalline (soft) and crystalline (hard) shells at the lowest organizational level, with thinning shells toward the exterior and an off-center hilum. At a higher level, blocklets are arranged along amorphous radial channels with smaller blocklets in the semicrystalline shells. The second-highest level reveals blocklets containing amorphous crystalline lamellae with amylopectin polymers depicted within them. The role of amylose–lipid and protein interactions in organizing amylopectin chains is highlighted.

## 4. Rice Starch Functional Features

Rice starch is a key molecule because of its unique features and qualities. Understanding these qualities helps to optimize different formulations. This overview focuses on how rice starch turns into a gel, expands, and stays stable, as well as its effects on texture.

### 4.1. Rice Starch Rheology

A material’s rheological properties refer to how it deforms and its flow patterns in response to stresses. The principal metric for determining rheological properties in starch is viscosity, which is applied as a thickener. Several studies have used rheological and gelatinization methodologies to determine these attributes [95,96,97]. The most widely used device for studying the viscoelastic nature of starch is the dynamic rheometer [98]. In characterizing gel, the storage and loss moduli (G’ and G″) are employed. G′ (storage modulus) can be used to compute the energy stored in the material and recovered during each cycle, whereas G′′ (loss modulus) quantifies the energy lost or dissipated during a sinusoidal deformation cycle. In the process of hot starch dispersing, both the storage modulus (G′) and loss modulus (G″) reach their peak values at a specific temperature, followed by a decline with continued heating. The initial increase in G″ is attributed to swelling due to amylose leaching, which allows the system to accommodate its full capacity [65,99]. Rice starch with higher amylose levels (18.86%) yields stronger gel structures, reflected in higher G′ (45.24 × 10^2^ Pa) and G″ (4.83 × 10^2^ Pa) values. Lower amylose content starch (7.83%) shows weaker gel properties with reduced G′ (26.30 × 10^2^ Pa) and G″ (3.50 × 10^2^ Pa) values. Weak internal organization and negatively charged phosphate groups in the starch granules also contribute to these rheological differences. [97]. According to [100], G′ readings for multiple forms of rice ranged from 53 × 10⁴ to 17.5 × 10⁴ Pa under heated conditions. The paste and thickener forms have the potential to be tested for rheological aspects, including the creep, flow behavior, mechanical bandwidth, viscoelasticity, as well as thickening power [16]. Measuring stress based on the applied acceleration rate can help evaluate the nature of flow [94]. The rheological behavior of rice starch reflects a balance between elastic (G′) and viscous (G″) properties influenced by its molecular composition gelatinization and retrogradation. Higher G’ linked to high amylose content indicates stronger gelation and a solid-like structure, while higher (G″) typical of low amylose or gelatinization reflects greater fluidity. Understanding these dynamics is essential for optimizing rice starch functionality in industrial applications.

### 4.2. Rice Starch Pasting Properties

**Figure 6:**
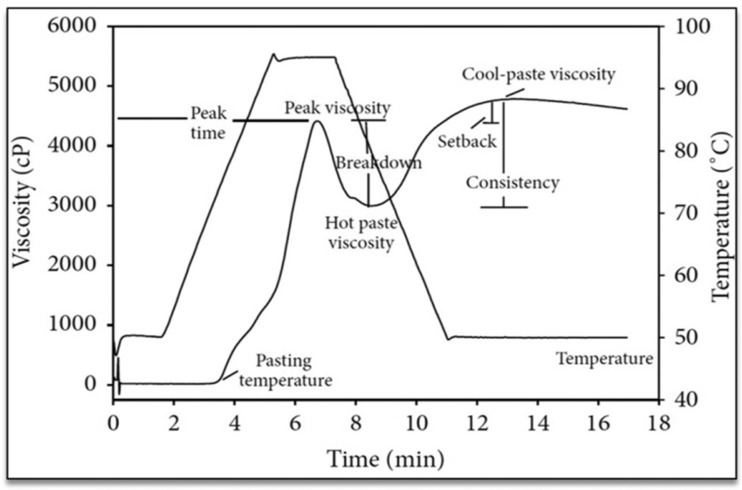
Characteristic pasting profile of starch as determined using Rapid Visco Analyzer (reprinted with the permission of Hindawi Kesarwani, Chiang, and Chen [105]).

Starch pasting properties describe its response to heat and water during processing, encompassing parameters such as peak viscosity, trough breakdown, final viscosity, and setback viscosity, which are measured with a Rapid Visco Analyzer (RVA) and rheometer apparatus. RVA analysis involves the meticulous preparation of milled rice samples, including grinding, sieving, and blending a defined amount with distilled water. Using the RVA, the temperature is cycled from 50 °C to 95 °C and back to 50 °C over approximately 17 min. Key viscosity metrics, including the peak, hot paste, cool paste, breakdown, setback, and consistency viscosities, are measured alongside the pasting temperature and peak time (see Figure 6). These properties offer crucial insights into starch’s cooking and processing performances, guiding its application across diverse industry sectors, functioning as a disintegrant, stabilizer, texturizer, and surface sizing and thickening agents [27,65,100,101]. Cooking starch in the presence of water leads to an absence of the crystalline domain and the expansion of its outermost layer. Starch’s warm pasting thickness is influenced by complex development (amylose–lipid), expanding, and leaching [102]. At the storage temperature, a mixture containing group acetyl in watery starchy dispersions reduces cohesion and discoloration by breaking links across amylopectin along with its outer links, plus amylase sequences. According to [74], modified starch exhibits greater pasting transparency over those that are natural. Heating starch in an excess of H_2_O causes amylose swelling, resulting in a thick paste. Pasting, which can occur before or after gelatinization, is a useful route to explore the functional characteristics and structural properties of starch. Its utility in commercial uses is defined by the thickened response and viscose conduct [103]. The G. Zag and K. Quder rice planted at extreme elevations of the Kashmir Valley with hardened freezing environments exhibits starch with higher amylose/amylopectin ratios. The SG displayed high pasting temperature (75–87 °C) and extreme time variations between 5.4 and 7.2 min. [91]. When rice starch was subjected to green tea polyphenolic compounds, the maximum temperatures and the enthalpy of gelatinization lowered, and this was owed to polyphenol–starch interlinking. Furthermore, tea polyphenolic compounds improved the starchy gel’s freeze–thaw resilience and hydrating qualities, preventing retrogradation [104].

### 4.3. Rice Starch Swelling Intensity

When the starch is boiled in an excess of H_2_O, H bonds break, disrupting its crystalline form. The H_2_O molecules establish H bonds with the exposed -OH groups of amylose/amylopectin. This causes enhanced solvability and swelling. The solvability and swelling intensity indicate the level of connection among starch sequences as well as within the SG glassy and crystal-like states [106]. Starch swelling precedes the elimination of dual refraction before solvability [65]. The cycle extent of this connection is controlled by the shaping arrangement of amylose or amylopectin, the SG contents, along with several important parameters [19]. Amylose–lipid interactions suppress swelling, whereas the swelling tendency of SG is predominantly dependent on the amylopectin configuration. Amylose, on the other hand, acts as a diluting agent in this process [42]. The swelling ability fluctuates with the temperature in both waxy and standard rice SGs. Standard rice SGs swell in two phases. Amylose has no effect on swelling during the first phase, which occurs around 55 and 85 °C. However, the existence of shorter amylopectin units (DP_n_ up to 9) increases the swelling capacity within 55–65 °C. Amylose lowers the swelling strength during the second phase, when temperatures range from 95 up to 125 °C. Shorter amylopectin sequences influence SG swelling in this initial phase, whereas amylose leaching affects the process in the second phase [107]. The swelling strength of starch is determined by its ability to retain water through hydrogen bonding. Once gelatinized, the hydrogen connections across cells of starch are destroyed and substituted with H_2_O/H bonds [108]. Techawipharat [109] investigated the swelling capacities of different waxy and common rice starches, finding that waxy starch presented a better swelling strength of 27% compared to ordinary rice starch, which was 15.5%. Granules of waxy rice starch demonstrated greater flexibility and were more prone to breakdown when swollen and densely packed [110]. Ordinary rice SGs were less prone to breakage, indicated less swelling, and appeared stiffer. Furthermore, very little leachate was seen from ordinary rice SGs, suggesting that amylose remained the predominant substance that was released within the granulation [111]. In contrast, the exudation from waxy rice SGs was substantial and principally composed of amylopectin [42].

### 4.4. Rice Starch Light Transmittance

Measuring light transmittance through starch means checking how much light goes through a starch solution or paste. This allows us to understand things like how thick it is, how clear it looks, and how strong its gel is. In food processing, like making gels/jellies and fruit pastes, having a clear starch paste is crucial. It helps ensure the right stability for finer quality goods [101]. Additionally, Gani [52] documented cloudy ratings ranging from 0.3% to 0.6%, illustrating the various starch architectures brought about by variations in pasting quality and turbidity. The clearness is influenced by factors such as starch origin, amylose/amylopectin, biological/chemical transformation, and the insertion of solutes.

It has been shown that the quality of the paste deteriorates with a reduced storage duration, both for native and processed starches. Compared to native starch, either oxidized or double-modified starches exhibited greater transmission percentages from diverse starch sources. This is primarily due to the organic replacement of carbonyl and carboxyl (-CO/-CO_2_H) groups with hydroxyl group (-OH). The paste clarity was reduced due to storage time in both native and modified starches. However, due to unsuitable gelatinization, decreased swelling activity, and the interconnected SGs, the paste produced poorer paste clarity than the native version, causing light to be scattered instead of mitigated by the resulting compact particles [112]. Rice starch exhibits high light transmittance due to a smaller granule size and low retrogradation, while starches from other source (corn, potatoes, etc.) show moderate to low transparency due to having larger granules and higher retrogradation or phosphate content.

### 4.5. Temperature and Gelatinization Features

Starch gelatinization transforms granular starch to a gel-like consistency through heating and water absorption. This process involves both amylose and amylopectin, with gelatinization starting at 60–70 °C and progressing up to 90 °C or higher. Elevated temperatures speed up gelatinization, thickening the gel as the starch granules swell. However, excessive heat can cause the gel to become too soft and watery. Cooling solidifies the gel, with texture influenced by both heating and cooling temperatures. Repeated heating and cooling can increase the gel density due to amylose retrogradation, underscoring the importance of temperature in determining the final texture.

The different thermal attributes of the starches that come from rice are illustrated in Table 4. Boiling starch in H_2_O induces the molecular and geometrical shifts within them, including swelling due to H_2_O absorption, amylose leaching into the H_2_O phase, and the disruption of the crystalline state caused by the breakup of amylopectin dual helices [113]. DSC, FT-IR, NMR, polarized light microscopy, thermomechanical analysis, as well as techniques like X-ray scattering are essential methods used to determine the starch gelatinization temperature [114,115,116]. The change in amylose phases; the form, shape, and placement of SGs; as well as the internal organization of segments inside the SG, cause such variations in the temperature during gelatinization [65]. The gelatinization temperature transition states (T_p_, T_c_, and T_o_) are also influenced by the crystallized state’s molecular configuration [117]. There exists a beneficial link between the amylose concentration and the gelatinization temperatures [118]. Type-A starches have a higher gelatinization temperature than type-B, and their enlarged temperature transition states are brought on by their advanced crystallinity form, which maintains their structural integrity and increases the SG resistance to gelatinization [119]. The term “Hgel” refers to the breakdown of the structural double-helical sequence, illustrating how the forces linking within amylopectin crystals form vary, resulting in double-helical sequences. This, in turn, leads to irregular meeting points of H bonds within molecules [120]. Several studies have confirmed the gelatinization capabilities of rice SGs [65,89,94,121]. Due to its higher water-binding capacity, inulin (a polysaccharide) increased all three gelatinization transition temperatures of rice SGs by approximately 3 °C when added [122]. The peak and final temperatures of rice starch were elevated when an additional electric field was applied, although these values decreased as the field strength increased [103].

## 5. Applications

Starch finds diverse applications across multiple sectors due to its functional properties. In the food industry, it serves as a thickener and stabilizer in products like sauces and gluten-free foods and helps maintain moisture and texture in baked goods such as cakes and bread. It also enhances the structural integrity and flavor of snacks, sweets, and processed foods. In construction, rice starch is utilized in eco-friendly adhesives and binders, leveraging its natural adhesive capabilities. In cosmetics, its absorbency and smooth texture make it essential for powders and creams. The textile industry employs rice starch as a sizing agent to improve fabric finishes and yarn quality. In paper production, it functions as a binder and coating agent, enhancing paper strength. In pharmaceuticals, rice starch acts as a binder and disintegrant in tablets, ensuring consistent drug release. Additionally, rice starch is crucial in the development of biodegradable plastics, providing a sustainable alternative to conventional materials.

### 5.1. Building Materials

Starch’s comparatively lower structural integrity and durability, compared to conventional building materials like concrete, steel, and wood, limit its primary use in construction. However, starch-based materials can serve as additives or binders in applications such as plasters, mortars, and adhesives. These materials may offer advantages like improved workability, adhesion, and moisture resistance. Ongoing research explores starch-based biodegradable alternatives for specific building needs, particularly in temporary and sustainable structures, aiming for environmentally friendly solutions.

Several studies proved that bio-based building materials have positive environmental impacts like carbon sequestration, biodegradability, reduction in energy consumption, and eco-friendly features [123,124,125,126]. In [127], five natural starch types, along with mortars containing clay minerals such as kaolinite and illite, were used to investigate the interactions between natural clays and starches. The samples were prepared with sand and clay dry-mixed at 70% and 30% in a weight percentage composition. The amount of starch used in preparation of the samples was 1% with 13.6% of water and 5% with 13.5% of water by dry weight composition of sand and clay. The mortar was thoroughly mixed at 62 rpm for 60 s, followed by an additional mix at 93 rpm for half a minute. Starch with a high amylopectin content notably enhanced the mechanical properties of kaolinite mortars. Comparative analysis revealed that rice starch formed stronger hydrogen bonds with kaolinite than maize starch, leading to superior structural reinforcement. Porosimetry confirmed that rice starch optimized the arrangement of grains and clay particles in the kaolinite matrix, enhancing its structural integrity. Molecular-level analysis revealed effective interaction between rice starch and clay [127]. The starch-based composites were synthesized by combining starches, including sticky rice starch, with sand, hemp shives, and water followed by compaction into molds and microwave heating to facilitate starch gelatinization. To improve water resistance, the StarchCrete samples were coated with paraffin and carnauba wax through immersion in melted wax. The hemp shive ratio significantly influenced the material’s properties, with an optimal ratio of 0.5 achieving the highest compressive strength of 2.8 MPa. Higher ratios resulted in reduced strength due to weaker bonding and an increase in hemp shives lowered both the density (from 1383 to 618 kg/m^3^) and thermal conductivity (from 0.52 to 0.15 W/(m·K)), enhancing insulation. Paraffin wax coating further enhanced water resistance and durability. Statistical analysis (ANOVA, *p* < 0.05) confirmed the significance of these effects, with StarchCrete outperforming other natural fiber composites in compressive strength, positioning it as a promising material for sustainable construction applications [128]. The combination of sticky rice and lime as primary binder with plant fiber as a supportive additive was used in diverse zones of China. In Shaanxi province, a higher proportion of waxy rice was incorporated into the binder mixture. Official buildings, signifying their importance and prestige, employed a binder containing up to 3.6% waxy rice. Domestic structures, while still benefiting from the binder’s properties, utilized a slightly lower concentration, up to 1.9%. Conversely, Shandong province demonstrated a different approach. Both official and domestic buildings employed a binder with a lower waxy rice content, reaching up to 1.2% and 1.3%, respectively. Mechanical tests showed better performance of mortar in lime with sticky rice. The crystal morphology analyzed using SEM indicated that a rich amount of sticky rice changes the irregular prismatic pattern, whereas H_2_O evaporation and internal transportation were seen in samples with a small quantity of sticky rice, resulting in greater porosity [129]. An amylose content of 21.5% in starch was used in mortar samples with a grain sand size up to 0.25 mm and natural wax. The starch samples were shown to be 30 MPa for compressive strength, with elastic moduli <2 GPa, a 0.20 MPa tensile strength, and good water-resistant capacity. The coating with high-molecular-weight compounds enhanced the durability of bio-based building materials [130]. The 65% starch binder solution and hemp were mixed properly and used in the preparation of prismatic and cubic samples. Hemp/starch composites exhibit a lower density compared to lime-based composites. This density difference is not proportional to the hemp shive content, indicating a non-linear relationship. This suggests a complex interplay between hemp shive incorporation and the resulting composite density, exceeding a simple linear correlation and tensile strength of 0.11 MPa. The stress/strain is linear, whereas Young’s modulus is non-linearly observed. Five percent hemp with starch binder enhanced the mechanical properties in this study [131]. In Gacoin et al.’s study [132], bio-based insulation materials were synthesized from viticulture byproducts (grape pomace, stalks, skins, and crushed stalks) combined with starch at a 20% mass ratio. The composites exhibited a moderate compressive strength (3.0–5.9 MPa), a Young’s modulus of 11.7–36 MPa, and a thermal conductivity of approximately 0.075 W/(m·K). The starch/grape pomace composite showed superior compressive strength. These grape pomace-based composites demonstrate promising mechanical and thermal properties, making them viable for insulation materials in public buildings [132]. Similarly, beet pulp- and starch-based composite building materials have shown good mechanical properties [133]. The mechanical properties of a NaCl–starch binder have shown improvements of 600% with 1% starch and 137% with up to 10% starch [134].

### 5.2. Cosmetics

Starch has a variety of functions in cosmetics, contributing to product texture and functionality. Its absorbent features make it exceptionally useful in formulations designed to control oil and moisture on the skin and hair. It is used for a variety of purposes, including for stabilizing/as a controller, dusting/bath powder, thickening, gel developing, and for adjusting sensory qualities [135]. Compounds originating from rice hold the ability to address skin-related issues. Extracts obtained from rice bran ashes not only enhance melanin production but also provide protection against UV-induced skin damage [136]. The positive consequences of an oil-in-water bath moisturizer were amplified by incorporating a zero-lipid bath composition containing a rice starch additive. Bathing in rice starch-infused water for fifteen minutes twice a day significantly boosted the healing ability of injured skin by 20%. Whenever powdered starch was incorporated into a bathing liquid, individuals with atopic dermatitis experienced enhancements in the integrity of their skin barrier [137]. The mixture of rice starch and moringa extract at a ratio of 3:1 was used in cream body scrub, and the findings reveal high antioxidant activity, physical valuation, spreading capacity, and pH, fulfilling the high standards of the product [138].

### 5.3. Textile Sector

Rice starch exhibits remarkable versatility in the textile industry, particularly in textile sizing, a crucial process in which yarns receive a protective coating before weaving. This coating enhances yarn strength, smoothness, and abrasion resistance during weaving. Moreover, it improves the weaving efficiency and fabric quality while also aligning with sustainable textile manufacturing practices due to its environmentally friendly nature. Additionally, rice starch finds application beyond sizing, being utilized in finishing treatments to impart desired fabric properties such as softness, wrinkle resistance, and moisture management [139,140]. Utilizing DORB (de-oiled rice bran) as both a fabric desizer and a component in cleaning soap has been proven effective. Optimal results were observed at 37 °C and pH 8.0 over a 96 h incubation period with 1.5% (*w*/*v*) maltose. The study indicates that the combined laundry power of detergents and enzymes surpasses their individual effectiveness, highlighting the necessity of α-amylase as a key ingredient in cleaning detergent formulations [141]. The starch was grafted with acrylamide with 2-(methacryloyloxy) ethanol to boost its resistance to abrasion and minimize hairy yarn appearance [3]. To create multifunctional textiles, the clothing industry is shifting toward sustainable manufacturing methods, aiming to reduce chemical usage, employ cost-effective equipment, streamline production processes, and minimize wastewater generation [142]. The advancement of functional textiles depends greatly on nanofinishing techniques and the application of biopolymer layers. For instance, to impart shrink-resistant features to wool clothing without compromising its inherent characteristics, coating it with biopolymers such as gums, chitosan, and starch has emerged as a promising sustainable alternative to energy-intensive treatments like UV rays and ozone [143]. The ZnO starch nanocomposites serve as active finishers, providing textiles with antibacterial and UV-shielding capabilities [3].

### 5.4. Paper Production

Starch is essential in numerous stages of the paper-making process, serving in binding, sizing, surface treatment, strength enhancement, and retention. Its versatility and effectiveness make it a highly valued additive in paper manufacturing, enhancing the overall quality of paper products [144]. The food sector ranks first in starch usage, amounting to USD 21.1 billion, while the paper industry follows closely behind [3]. By impeding the capillary action in fibers to absorb fluids, a sizing agent diminishes the fluid absorption of dry paper, resulting in precise, cost-effective, and uniform printing surfaces. Moreover, sizing influences the surface-bonding capacity, uniformity, printer compatibility, and minimizes roughness and fuzzing while also decreasing interface porosity. However, the insoluble features of natural starch in most liquids at normal temperatures and its inadequate H_2_O barrier characteristics limit its application in the paper synthesis system [145]. Modified starches play a crucial role in surface sizing solutions, improving paper’s smoothness, water repellency, and strength. Specifically, amphiphilic starch materials containing both hydrophilic and hydrophobic groups interact with paper fibers, forming a coating that enhances water repellency.

As far as binders are concerned, they are like glue for pigment particles on paper, sticking them together and to the paper’s coating. After pigments, they’re the second most common component for soft-tinted paper [14]. Colorful papers use two forms of binder: one helps keep the coating smooth and holds water, while the other does most of the sticking. The main binder is made from synthetic material, while the helper binder is made from bio-based materials like starch [146]. The intractable nature of natural starch and its reduced viscosity after boiling and storage make it unsuitable for use as a binder. Moreover, upon exposure to water during production, starch infiltrates the paper matrix, resulting in binder migration and significant alterations in the porosity of the paper [147], while modified starches can be tailored for specific applications in paper synthesis by altering their molecular structure [148]. The positively charged (cationic starch) form of modified starch is used to enhance fiber bonding in paper. It is especially useful for increasing retention of fine particles and fillers during the paper-making process and the chemically altered (oxidized starch) to increase its solubility and make it more suitable for surface sizing and coatings. It provides excellent film-forming properties, leading to a smoother surface finish.

### 5.5. Bio-Based Plastics

Rice starch is widely employed in bioplastic forming as both a biodegradable filler and a reinforcing agent. When combined with biopolymer matrices, starch-based bioplastics present superior mechanical properties, biodegradability, and sustainability compared to conventional petroleum-based plastics.

Rice starch enhances the flexibility, moisture resistance, and processability of bioplastic materials, expanding their utility across diverse sectors including packaging or wrapping. Overall, rice starch plays a crucial role in driving the development of eco-friendly bioplastic alternatives [149]. The rice containing starch and glycerol mixed at a high temperature present better tensile power and viscoelasticity of the derived bioplastic with heat treatment [150]. Rice flour containing 93% starch is a promising candidate for bio-based plastics. By cross-linking starch with sodium trimetaphosphate (at a 1–3% concentration) along with a plasticizer, improved biodegradability and tensile capacity (4.3 MPa) have been demonstrated [151]. Adding oxidized cellulose improved the strength of the cellulose–starch-based bioplastic. This happened because of more −COO− groups and less crystallinity. It shows that the properties of bioplastics are greatly affected by their functionality [149].

### 5.6. Pharmaceuticals

Rice starch is widely used in the pharmaceutical industry as an excipient in tablet preparations, serving as a binder, disintegrant, and filler. It binds tablet ingredients together for uniformity and cohesion, facilitates tablet dissolution and drug release in the gastrointestinal tract, and adds bulk to tablets for compression. Rice starch’s inert nature, affordability, and compatibility with active pharmaceutical ingredients make it a popular choice for pharmaceutical formulation [3]. Cadexomer iodine is a commercially available starchy product composed of a three-dimensional structure of cross-linked hydrophilic starch composite. The product soaks up fluid and debris from the injury, releases iodine to cleanse it, and shows resistance against bacteria [152]. Starch is increasingly utilized in bone tissue composite production due to its low cost, compatibility with biological systems, abundance of hydroxyl groups for bonding to hydroxyapatite (HA), and the ability to modify it to meet specific composite production requirements. Scientists have studied mixing hydroxyapatite (HA) with starch to create an effective replacement [153]. Due to the accessibility of starch hydroxyl groups, natural starch can be employed in the development of hybrid printed products. By combining starch with gelatin nanoparticles and biological collagen, a 3D printed bio-ink was successfully created [154].

### 5.7. Bakery and Dairy Items

Starch plays a crucial role in bread preparation, absorbing approximately 64% of the liquid used. Additionally, it serves as an inert additive within the consistent protein structure during the maturation of the dough. Throughout the dough’s fermentation process, it functions as a continuous network of both carbohydrates and proteins. Moreover, the interaction between wet gluten and aggregating SGs influences the rheological properties of the dough. Furthermore, granule morphologies may also play a role in retaining gas bubbles within the dough. Gas bubbles could be supported by a tiny granule, but they are destabilized by granules bigger than the gas walls of the cells. Additionally, during preservation, retrogradation impacts the bread’s texture and quality [155]. Similar to bread, the traits of biscuits are greatly affected when cross-linked carbohydrates are substituted [156]. Upon baking, the cross-linking process increased the heat strength, decreased the viscosity, and decreased the biscuit’s weight along with raising its stiffness [157]. Further research has been conducted on using starch as a substitute for fat in baked goods [158]. The study found that altering the levels of substituted cross-linked starch negatively affected both the growth and durability of the dough. However, an 8% starch replacement had no appreciable effect on the muffins’ baking characteristics, such as the weight loss, specific volume, moisture levels in the crumb, or coloring metrics. Additionally, muffins containing an 8% substitution of starch displayed high acceptance ratings and textural properties that were comparable to those of the standard recipe [157]. Dairy-based goods contain starch, which serves as a stabilizer and imparts a smooth consistency and taste. It is being investigated extensively for use as a thickener in yogurt production to give milk curd a more palatable texture and to lessen apparent flaws and ruptures [159]. The addition of acetylated starch demonstrably increased yogurt firmness and imparted a finer, more homogeneous microstructure. Furthermore, the incorporation of acetylated starch improved the flow characteristics of the yogurt. Moreover, the hydrogen bonding between proteins and starches could enhance the rheological properties of yogurt containing starch [160]. Ice cream, characterized by its significant fat content, is commonly derived from milk. The effects of incorporating varying amounts of cross-linked citric starch, up to 2%, into ice cream with different fat concentrations were studied. The study participants found that the inclusion of 1% starch resulted in notable sensory improvements and enhanced the texture of ice cream, making it the texture of standard ice cream (containing 11% fat). Increasing the starchy content improved the firmness and freezing–thawing durability of the ice cream, while substituting 5% fat helped retain its smoothness [161].

### 5.8. Processed Food

Rice starch is widely used in meat and food processing for its versatile and adaptable properties as a binder, extender/filler, fat substitute, yield improver, moisture retainer, and emulsifier [162]. Heat-induced expansion of SGs in the protein-based hydrogel matrix is potentially favorable in meat emulsions. Moreover, in the process of gelatinization, SGs will consume liquid, improving the meat item’s capacity to retain water [163]. When starch is heated with protein (myofibrillar), the SGs spread and gelatinize, filling the protein networks that contain myofibrillar. Water adheres to the blend, contributing to the formation of the texture of the myofibrillar gel protein [164]. The incorporation of starch can enhance the water-binding capacity of meat patties. The introduction of starch influenced the water-binding properties of pig patties, resulting in improved shear values, which, in turn, reduced the thawing loss and enhanced the binding capacity to water.

### 5.9. Challenges and Future Directions

Despite rice starch being used in many applications because of its chemical characteristics and related properties (Table 5), it still presents several challenges for industrial uses, which are due to its low amylose content, controlled gelling properties, and sensitivity to retrogradation. To address these issues, future innovations can involve genetic and innovative modification techniques and enzyme treatments to enhance its functional properties. Additionally, it has the potential for sustainable applications (green chemistry and biodegradable materials). However, significant research gaps persist in optimizing the extraction methods, modification techniques, and exploring emerging applications. Overcoming these challenges will enhance the versatility of starch and broaden its potential across various industrial sectors.

## 6. Conclusions

Starch constitutes nearly ninety percent of the total mass of grains, serving as the primary component in rice. Despite appearing chemically straightforward, starch stands out as one of the most intricate carbohydrates, marked by numerous unresolved investigations regarding its metabolism, core structure, and the interplay between its structure and physical features. Amylose is a key component of rice starch along with amylopectin. Starch is found in a particular semicrystalline form with the thinnest SG (2 to 7 μm). Starch granules typically exhibit a polyhedral shape with sharp edges and can feature either asymmetrical or smooth patterns. Although the popular technique for separating rice starch is alkaline extraction, it produces an extremely saturated alkaline sewage. As an alternative, high-purity starch is separated using enzymatic and various physical techniques, avoiding the release of harmful byproducts. Starch is insoluble in cold water, but upon cooking in an aqueous solution, the starch granules expand, and hydrophilic segments leach out, undergoing a phase transition toward gelatinization. The features of rice starch are changed by the proportion of amylose to amylopectin and also by the branched patterns of the amylopectin. After hydroxypropylation, native starch demonstrates enhanced swelling and solubility, but its viscosity decreases, while cross-linked starches exhibit reduced expansion and dissolution, indicating improved pasting attributes. Conversely, dual-modified starches offer superior attributes compared to native starches, with the order of dual modifications also influencing the starch’s traits. Chemically treated starches are well suited for a broad spectrum of commercial applications. Nonetheless, their standards must be elevated to facilitate their flexible functionality within the food sector, such as sugar coating, as well as serving as thickening agents, fat replacements, and pharmaceuticals supplements. In non-food sectors, they are used in cosmetics, as building material additives, plastic items, and in paper production before utilization. Future research on rice starch should focus on advancing modification techniques and sustainable methods. Investigating novel applications in emerging fields like nanotechnology and optimizing extraction processes will significantly expand its industrial potential.

## Figures and Tables

**Table 1:** Features of amylopectin identified among multiple kinds of rice [1,43,46].

Characteristics	Indica	Japonica Waxy
CL (average)	21–22	19–20
DP_n_ (10^3^)	4.7–5.8	8.2–12.8
F1	85–130	120–180
F2	42–44	41–44
F3	16–17	16–17
Iodine capability (g/100 g)	1.62–2.57	0.39–0.87
P (glu-6-PO4). (μg/g)	9–28	8–13
P (all) (μg/g)	11–29	8–13
Beta-amylolysis (%)	56–59	58–59

**Table 2:** Rice starch chemical compositions.

Sample	Protein %	Amylose %	Fat %	Origin	Reference
Arborio	-	14.1	-	Italy	[58]
Yuzhenxiang	-	17.2	-	China	[59]
Guangyou 2928	-	26.3	-	China	[60]
Dodamssal	-	42.8	-	Korea	[61]
Shinkiari NTHRI	-	<20	-	Pakistan	[62]
Mushkbudji K448	0.23	30.2	0.33	India	[52]
Kohsar	0.52	6.3	0.1	India	[50]
Mavr	-	24	-	Brazil	[63]

**Table 3:** Rice starch geometry, appearance, and dimension in granular form.

Appearance and Geometry	Dimension (µm)	Reference
Polyhedral (PH), uneven, and sharp edges	3.9 to 4.5	[89]
PH	4.0 to 5.2	[52]
PH and asymmetrical	1.0 to 8.0	[94]
PH and uneven	1.5 to 6.1	[16]
PH and irregular	1.5 to 5.8	[75]
PH and smooth pattern	1.53 to 6.7	[91]

**Table 4:** Rice starch thermal features.

Proportion (wt:wt) H_2_O: Starch	Heating Speed (°C·min^−1^)	T_c_ (°C)	T_o_ (°C)	T_p_ (°C)	H_gel_ (J/g)	Reference
3:1	10.00	71–79	56–64	61–75	9–13	[89]
3:1	10.00	147–156	37–40	61–74	24–29	[16]
2:1	10.00	74–78	66–67	61–74	8–12	[65]
3:1	10.00	83–90	59–62	61–74	11–19	[94]
2:1	10.00	72–76	60–63	65–68	10–11	[121]
2:1	10.00	62.4	84.4	74.57	9.43	[122]

T_c_ = conclusion gelatinization temperature; T_o_ = onset gelatinization temperature; T_p_ = peak onset gelatinization temperature; H_gel_ = gelatinization enthalpy.

**Table 5:** Summary of chemical, physical, and structural properties of rice starch along with its applications.

Features	Remarks	References
Chemical composition	Rice starch is primarily composed of amylose (linear polymer) and amylopectin (branched polymer). The ratio of these components affects properties like its gelatinization, pasting features, swelling properties, rheology, and light transmittance behavior.	[22,33,34,35,36,37,38]
Granule structure	It is characterized by small granules (2–7 μm) with polyhedral (PH) shapes.	[22,90]
Gelatinization	Gelatinization occurs between 60 and 90 °C or higher, involving water absorption, granule swelling, and amylose leaching. Amylose content and crystallinity affect the gelatinization temperature and texture.	[65,117,120,121,122,123]
Swelling properties	Starch granules swell upon heating in water, breaking hydrogen bonds and forming new ones with water molecules. Waxy rice starch has a higher swelling capacity than ordinary rice starch. Amylose acts as a diluting agent and suppresses swelling in later phases.	[42,106,108,109,110,111]
Pasting features	Rice starch responds to heat and water during processing. Key parameters include the peak viscosity, breakdown, final viscosity, and setbacks. It is influenced by amylose–lipid complexes, starch modifications, and additives like polyphenols. It is known for having high paste clarity and stability. These properties influence starch’s functionality in applications such as thickening, stabilizing, and texturizing across various industries.	[27,65,74,100,101,102]
Rheology	Rice starch exhibits viscoelastic behavior. The storage modulus (G′) reflects elastic properties, and the loss modulus (G″) represents viscous dissipation. Higher amylose content enhances G′, indicating stronger gelation.	[94,97,100]
Light transmittance	High light transmittance is due to its small granule size. Chemical modifications improve transparency, while storage reduces clarity. It is influenced by the amylose/amylopectin ratio and starch origin.	[52,101,112]
Modifications	Physical, chemical, and enzymatic modifications enhance specific properties. Cross-linking enhances its resistance to heat, shear, and acids. Hydroxypropylation improves solubility, hydrophilicity, and freeze–thaw stability. Oxidation and dual modifications improve stability and clarity.	[72,73,77,80,82,86,87]
Applications	Building materials: It is used in plasters, mortars, and adhesives; biodegradable options improve sustainable construction. Rice starch enhances mechanical strength, while hemp/starch composites insulate effectively. Wax coatings boost durability. Cosmetics: It controls oil and moisture as an absorbent, stabilizer, and thickener, enhancing skin care. Textiles: It strengthens yarns and improves abrasion resistance; starch nanocomposites add antibacterial properties. It supports sustainable production by reducing chemical use with UV shielding. Paper production: It binds, strengthens, and sizes paper; modified starches enhance smoothness and bonding. Cationic and amphiphilic variants offer advanced water repellent properties. Bio-based plastics: It enhances biodegradability and flexibility; cross-linking boosts mechanical properties. It serves as a filler and reinforcing agent for sustainable plastic innovations. Pharmaceuticals: It functions as a binder and filler in tablets and supports bone tissue with composites. Cadexomer iodine aids wound healing; it maintains its integrity in medical applications. Food industry: It thickens, stabilizes, and emulsifies in processed foods; cross-linked starch improves texture. Gelatinization ensures water retention and quality in meat and dairy products.	[3,127,128,129,130,131,132,133,134,135,136,137,138,139,140,141,142,143,144,145,146,147,148,149,150,151,152,153,154,155,156,157,158,159,160,161,162,163,164]
